# Systems-Based Approaches to Cardiometabolic and Chronic Disease Management in Adult Clinical Practice: A Systematic Review

**DOI:** 10.7759/cureus.111024

**Published:** 2026-06-17

**Authors:** Anand Sekar G, Niveda Ashokkumar, Avijit Saha, Raghunandan D, Hariballav Mahapatra, Hairya Ajaykumar Lakhani

**Affiliations:** 1 Department of Cardiology, Aarupadai Veedu Medical College, Vinayaka Missions Research Foundation (VMRF-DU), Puducherry, IND; 2 Department of General Medicine, Meenakshi Medical College Hospital and Research Institute, Kanchipuram, IND; 3 Department of General Medicine, The West Bengal University of Health Sciences, Kolkata, IND; 4 Department of Medicine and Surgery, Indira Gandhi Medical College and Research Institute, Puducherry, IND; 5 Department of Diabetology, Sevayan Diabetes Centre, Puri Shree Hospitals, Bhubaneswar, IND; 6 Department of Internal Medicine, Smt. B. K. Shah Medical Institute and Research Centre, Vadodara, IND

**Keywords:** cardiometabolic disease, chronic disease management, integrated care, multidisciplinary care, telemonitoring

## Abstract

Cardiometabolic and chronic diseases frequently coexist in adult clinical practice, creating complex care needs across hypertension, type 2 diabetes, cardiovascular disease, chronic kidney disease, dyslipidemia, obesity, and multimorbidity. This systematic review evaluated systems-based approaches for adult cardiometabolic and chronic disease management, including integrated care, multidisciplinary teams, chronic care management, shared medical appointments, telemonitoring, pharmacist- and nurse-supported care, and technology-assisted models. A structured search identified 252 records; after duplicate removal, title and abstract screening, and full-text eligibility assessment, 11 studies were included. Because the studies differed in design, setting, population, intervention components, follow-up duration, and outcome reporting, findings were synthesised narratively. The strongest and most consistent effects were observed for intermediate clinical outcomes, especially systolic blood pressure and HbA1c, particularly when interventions incorporated structured monitoring, medication optimisation, patient education, multidisciplinary input, and coordinated follow-up. Several studies also reported improvements in quality of life, anxiety symptoms, medication safety, provider workload, reimbursement feasibility, and care coordination. Findings for lipid control, body mass index, behavioural outcomes, and major cardiovascular, renal, or mortality endpoints were less consistent. Risk of bias varied, with randomised trials generally showing fewer concerns than retrospective, nonrandomized, and quality-improvement studies. Overall, systems-based care may improve risk-factor control and care processes in adults with cardiometabolic multimorbidity, but larger pragmatic trials with longer follow-up are needed to determine whether these benefits translate into durable reductions in complications, hospitalisation, and mortality.

## Introduction and background

Cardiometabolic and chronic diseases are major concerns in adult clinical practice because they are common, progressive, and frequently coexist in the same patient. Cardiovascular diseases are the leading cause of death globally, accounting for an estimated 19.8 million deaths in 2022 and representing approximately 32% of all global deaths [1,2]. Cardiometabolic risk is closely linked with population ageing, hypertension, obesity, diabetes, dyslipidemia, chronic kidney disease, and modifiable behavioural and environmental factors [2]. These overlapping conditions contribute to multimorbidity and require simultaneous attention to blood pressure, glycemic control, lipid management, renal function, weight, medication burden, lifestyle modification, and psychosocial support. This complexity highlights the limitations of single-disease care models and supports the need for systems-based approaches in adult chronic disease management [3].

Chronic disease care is often delivered by individual diagnosis, through separate clinical specialities, and within short clinic visits. This model may be inadequate for adults with cardiometabolic multimorbidity because they require ongoing monitoring, timely treatment escalation, coordinated follow-up across multiple clinical fields, medication review, and shared decision-making. Effective pharmacological management and monitoring are recommended for adult patients with high blood pressure [3]. Patient-centred care, population diabetes care, team-based diabetes care, and timely therapeutic decision-making are also emphasised in current diabetes standards [4]. Risk stratification, longitudinal monitoring, and standardised terminology for kidney disease are important for consistent chronic kidney disease assessment and reporting [5], while coordinated management of kidney disease and cardiovascular-metabolic risk is supported by current chronic kidney disease guidance [2].

Fragmentation in chronic disease care has led to the evolution of systems-based approaches. For this review, systems-based care is defined as a redesign of routine care using a multidisciplinary team, integrated care pathways, structured follow-up, telemonitoring, shared medical appointments, medication review with the pharmacist, nurse-led coordination, chronic care management, behavioural support and/or technology-assisted monitoring. Multimorbidity integrated care models attempt to promote continuity, engagement, self-management, and communication among providers in health care [6] but are not uniform in structure or effectiveness. The review therefore draws on both integrated-care literature and relevant disease-specific guidance to contextualise systems-based management across cardiometabolic and chronic disease care [4].

Recent clinical studies demonstrate the range of systems-based strategies used in adult cardiometabolic and chronic disease care. Integrated heart-nephrology-diabetes care has been studied in patients with complex cardiovascular disease, diabetes, and chronic kidney disease as a model for managing multimorbidity as an interconnected condition rather than as isolated diseases [7]. Technology-assisted diabetes programs using structured assessment, customised reports, risk stratification, and nurse follow-up have been shown to improve achievement of cardiometabolic targets in patients with type 2 diabetes [8]. Integrated primary care cardiometabolic risk programs have also been explored for improving clinical outcomes and patient engagement through collaboration between interdisciplinary teams and primary care physicians [9].

Other systems-based models have focused on quality of life, behavioural support, and primary care redesign. Chronic Care Model interventions involving pharmacists, nurses, dietitians, and general practitioners have been evaluated for health-related quality of life among adults with hypertension, diabetes, and hyperlipidemia [10]. Nurse-led behavioural management has been evaluated in community-based care for adults with diabetes and hypertension [11]. Team-based hypertension care has also been studied using blood pressure reassessment, medication-adherence review, lifestyle counselling, and physician feedback [12].

Within systems-based cardiometabolic care, patient-centred, culturally responsive, social determinant-informed, and group-based approaches are also relevant. Integrated care interventions have addressed social determinants of health by combining medical management, behavioural health support, care coordination, diabetes education, culturally responsive counselling, language-appropriate communication, and attention to psychosocial needs, health literacy, access barriers, and community context among Latino adults with type 2 diabetes [13]. The Chronic Care Model has been applied to adults with diabetes and/or high cardiovascular risk through shared medical appointments that integrate group education, multiple disciplines, and self-management planning [14]. Telemonitoring models extend chronic disease care beyond the clinic by using blood pressure and glucose data to prompt additional monitoring or clinical intervention [15].

Other drivers to embrace systems-based care include medication optimisation, cost-effectiveness, and health-system sustainability. Expanded chronic care management programs involving pharmacists and community health workers can improve medication safety, reduce provider burden, decrease avoidable service use, support task-sharing, and strengthen long-term program sustainability [16]. Cardiometabolic clinic models have also been developed to address gaps between specialities and improve medication optimisation by combining expertise from endocrinology, pharmacy, and cardiovascular care [17]. Lifestyle support is an important adjunct to systems-based cardiometabolic care, as physical activity and exercise have established roles in reducing cardiometabolic risk and improving chronic disease outcomes [18].

The evidence remains heterogeneous by study design, population, intervention intensity, clinical setting, follow-up duration, and outcome selection. Some studies focus on systolic blood pressure (SBP) or HbA1c, whereas others evaluate quality of life, medication optimisation, care coordination, provider workload, reimbursement feasibility, cardiovascular outcomes, or renal outcomes. This variation limits clear interpretation of the overall contribution of systems-based care to adult chronic disease management. This systematic review evaluates systems-based approaches for adult cardiometabolic and chronic disease management and summarises their effects across clinical, patient-centred, medication-related, and health-system outcomes.

Objectives of the review

This systematic review aimed to explore the effectiveness of systems-based interventions in the management of cardiometabolic and chronic disease in adults in clinical practice. They evaluated their effect on clinical outcomes, including blood pressure, glycemic control, lipid outcomes, BMI, cardiovascular events, renal events, and patient-centred, medication-related and health-system outcomes such as quality of life, medication optimisation, care coordination, provider workload and sustainability.

## Review

Methodology

Study Design

This systematic review was undertaken to assess the evidence for systems-based approaches in adult clinical practice for the management of cardiometabolic and chronic disease. For this review, systems-based approaches were defined as care-delivery models that affected routine clinical practice and extended beyond standard clinician-patient interactions. These models included multidisciplinary team care, integrated care pathways, chronic care management, telemonitoring, shared medical appointments, pharmacist- or nurse-supported care, technology-assisted care, and coordinated service delivery. The review process was structured according to Preferred Reporting Items for Systematic Reviews and Meta-Analyses (PRISMA) guidelines for study identification, screening, eligibility assessment, and inclusion. No review protocol was registered.

Search Strategy

A systematic literature search was performed to identify studies that assessed systems-based care models for managing cardiometabolic and chronic disease in adults. The search was conducted using PubMed/MEDLINE, Google Scholar, Cochrane Library, and manual screening of reference lists from eligible full-text articles. The search included studies published from January 2007 through December 2025, spanning the publication period of the studies included in this review. Search terms were developed around three core concepts: cardiometabolic or chronic disease, adult clinical care, and systems-based intervention models. Search terms included combinations of the following: cardiometabolic disease, chronic disease management, type 2 diabetes, hypertension, cardiovascular disease, chronic kidney disease, integrated care, team-based care, multidisciplinary care, chronic care model, telemonitoring, shared medical appointments, care coordination, cardiometabolic clinic, pharmacist-led care, nurse-led care, and primary care. Boolean operators were used to combine search terms. An example search string was: “cardiometabolic disease” OR diabetes OR hypertension OR “chronic disease” AND “integrated care” OR “team-based care” OR “chronic care model” OR telemonitoring OR “shared medical appointment” AND adults. The search strategy was designed to capture both recent studies and foundational literature relevant to integrated care, chronic care management, telemonitoring, and cardio-renal-metabolic (C-R-M) care delivery.

Eligibility Criteria

Studies were included if they involved adult patients aged 18 years or older and assessed patients with cardiometabolic or chronic diseases, including hypertension, type 2 diabetes, cardiovascular disease, chronic kidney disease, dyslipidaemia, obesity, thyroid disease, autoimmune disease, or multimorbidity. The selected studies evaluated a systems-based approach to care delivery within an adult clinical practice setting and measured at least one clinically relevant, patient-centred, medication-focused, or health-system-focused outcome. Studies were excluded if they were not related to adult clinical practice, did not assess a systems-based care model, had non-extractable outcome data, were not published as full-text articles, or were published in a language other than English. Studies were also excluded if they did not include a care-delivery or systems-level component.

Study Selection

Records identified through the literature search were screened sequentially. Duplicate records were removed before title and abstract screening. Titles and abstracts were screened to exclude studies that were clearly irrelevant to the review question. Full-text articles were then assessed for eligibility. Eligibility was determined according to the study population, intervention model, clinical setting, and reported outcomes in relation to the scope of the review.

Data Extraction

Data were abstracted from each study included in the review, using a common evidence table. The information extracted consisted of the author(s), publication year, country, clinical setting, design of the study, number of subjects in the study, the population studied, type of intervention, length of follow-up, assessment of outcomes, main findings, direction of effect, and most important limitations of the main study. Where possible, numerical outcome data were abstracted, including for SBP, HbA1c, lipid outcomes, BMI, medication optimisation, quality of life measures and health-system outcomes. If the results were presented narratively or as target attainment instead of absolute change, then the results were summarised descriptively.

Outcomes of Interest

The outcomes were grouped into four categories: clinical outcomes, patient-centred outcomes, medication-related outcomes, and health-system outcomes. Clinical outcomes included blood pressure, glycaemic control, lipid parameters, body mass index, cardiovascular events, renal events, mortality-related events, and disease-control endpoints. Patient-centred outcomes included health-related quality of life, anxiety symptoms, depression symptoms, patient engagement, and self-management. Medication-related outcomes included medication optimisation, initiation of guideline-directed therapies, adherence support, and identification of potential adverse drug events. Health-system outcomes included care coordination, provider workload, reimbursement feasibility, program participation, and sustainability of care models. The outcome categories and corresponding measures assessed in the review are summarised in Table 1.

**Table 1:** Outcome Measures Assessed SBP: systolic blood pressure, DBP: diastolic blood pressure

Outcome category	Measures assessed
Clinical outcomes	SBP, DBP, HbA1c, lipid panel, BMI, cardiovascular events, renal events, mortality-related events, and disease-control endpoints
Patient-centred outcomes	Health-related quality of life, anxiety symptoms, depression symptoms, patient engagement, and self-management
Medication-related outcomes	Medication optimisation, initiation of guideline-directed therapies, adherence support, and identification of potential adverse drug events
Health-system outcomes	Care coordination, provider workload, reimbursement feasibility, program participation, and sustainability of care models

Risk of Bias Assessment

Design-specific tools were used to assess the risk of bias. The Cochrane Risk of Bias 2 (RoB 2) [19] tool was used to assess the randomised controlled trials and to assess the risk of bias in the randomisation process, deviation from intended interventions, missing outcome data, outcome measurement and selection of reported results. The Risk of Bias in Non-randomised Studies of Interventions tool (ROBINS-I) [20] was used to evaluate the risk of bias from the following areas for nonrandomized, retrospective, implementation, and quality-improvement studies: bias from confounding, selection of participants, classification of interventions, deviation from intended interventions, missing data, measurement of outcomes, and selection of reported results. An overall risk-of-bias judgment was drawn for each study based on the assessment of the domains of the study. Randomised trials were classified as low risk, some concerns or high risk of bias based on RoB 2 guidance. Nonrandomized studies were given a low, moderate, serious or critical risk of bias based on ROBINS-I guidance, which were presented as low, moderate or high risk for consistency in the presentation. Randomised trials were usually less prone to selection bias, but involved delivery of interventions in an open-label format, some likely attrition, and small numbers or incomplete outcome data. Nonrandomized studies and quality improvement studies tended to be of higher risk due to the potential for confounding, convenience sampling, selection bias, and lack of blinding. Moderate-to-high bias ratings were mainly due to real-world implementation designs, non-randomised methods, possible confounding, and lack of blinding; these factors were considered during evidence interpretation.

Data Synthesis

Given the differences in study design, population, type of intervention, clinical setting, outcome definition, follow-up period, and outcome reporting among studies included, a narrative synthesis was conducted. No attempt at direct quantitative pooling was made, and so a meta-analysis was not performed because of these clinical and methodological differences. Results were summarised under the two major outcome themes. The first theme reviewed clinical outcomes with the use of interventions across systems with a focus on SBP and glycemia. The second theme focused on patient-centred, medication-related and health system outcomes such as quality of life, medication optimisation, care coordination, provider workload and feasibility of reimbursement. Data presented in numerical form were summarised in tables and figures wherever possible to facilitate comparisons with other studies. Decreases in the values of SBP and HbA1c were interpreted as an improvement in the intervention group versus the control or usual care group, with negative values indicating a larger improvement. The studies included in the analysis were conducted in different designs and measured different outcomes, so interpretations of findings were based on direction of effect, statistically significant results, characteristics of the interventions, and risk-of-bias assessments.

Results

Search Results

**Figure 1:**
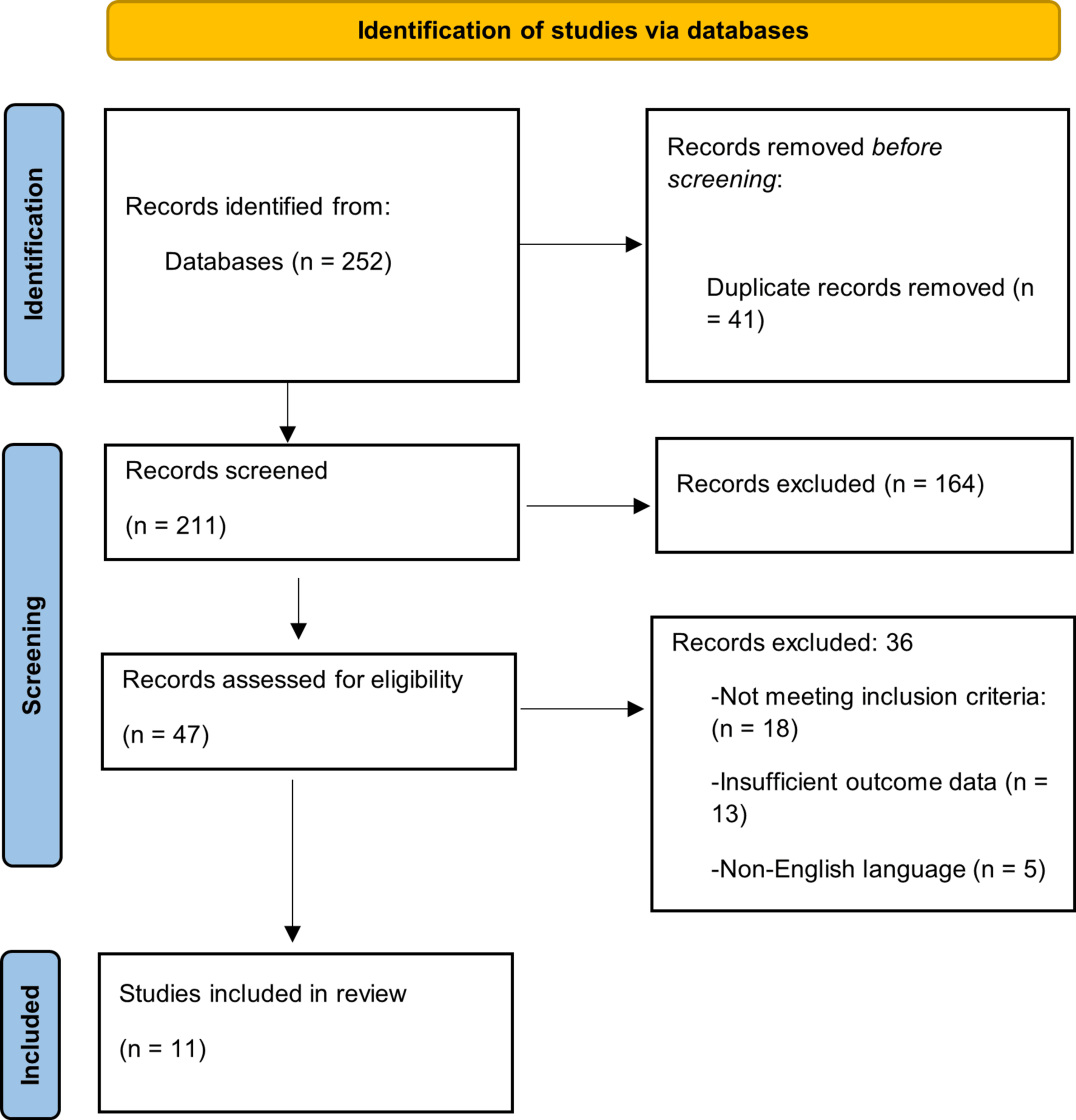
Preferred Reporting Items for Systematic Reviews and Meta-Analyses (PRISMA) flow diagram

A total of 252 records were identified through the database search. After removal of 41 duplicates, 211 records were screened by title and abstract. Of these 211 screened records, 164 were excluded as not relevant to the review question. The remaining 47 full-text articles were assessed for eligibility. Thirty-six full-text articles were excluded, including 18 that did not meet the inclusion criteria, 13 with insufficient outcome data, and five that were not published in English. Eleven studies were included in the final review. The study selection process is summarised in Figure 1.

Study Characteristics

The 11 included studies evaluated systems-based approaches to cardiometabolic and chronic disease management in adult clinical practice. The included interventions consisted of team-based care, nurse-led case management, Chronic Care Model programs, shared medical appointments, integrated cardiometabolic clinics, mHealth telemonitoring, culturally tailored integrated care, technology-assisted diabetes care, and expanded chronic care management teams. Study characteristics and key findings are summarised in Table 2.

**Table 2:** Characteristics and key findings of the 11 studies included CKD: Chronic Kidney Disease, MARCE: Major Adverse Renal and Cardiovascular Events, HRQoL: Health-Related Quality of Life, NT-proBNP: N-terminal pro–B-type Natriuretic Peptide, JADE: Joint Asia Diabetes Evaluation, HbA1c: Hemoglobin A1c / Glycated Hemoglobin, BP: Blood Pressure, LDL-C: Low-Density Lipoprotein Cholesterol, A1c: Hemoglobin A1c / Glycated Hemoglobin, EQ-5D: EuroQol Five-Dimension questionnaire, EQ-VAS: EuroQol Visual Analogue Scale, QoL: Quality of Life, SBP: Systolic Blood Pressure, DBP: Diastolic Blood Pressure, ABPM: Ambulatory Blood Pressure Monitoring, mmHg: Millimetres of Mercury, VA: Veterans Affairs, mHealth: Mobile Health, BMI: Body Mass Index, GFR: Glomerular Filtration Rate, CCM: Chronic Care Management, GLP-1 RA: Glucagon-Like Peptide-1 Receptor Agonist, SGLT2 inhibitors: Sodium-Glucose Cotransporter-2 Inhibitors, eGFR: Estimated Glomerular Filtration Rate

Study	Country/setting	Study design	Sample size	Population	Systems-based approach	Follow-up	Outcomes assessed	Key findings	Direction of effect	Main limitation
Evén et al., 2024 [7]	Sweden; integrated heart–nephrology–diabetes clinic	Prospective randomised open-blinded endpoint trial	131	Adults with cardiovascular disease, diabetes, and CKD stages 3–4	Integrated, person-centred multidisciplinary care involving cardiology, nephrology, endocrinology, and specialist nurses	2.0–5.7 years	MARCE, heart-failure hospitalisation, HRQoL	Integrated care did not significantly reduce MARCE compared with traditional specialist care, but improved several HRQoL dimensions and showed a trend toward fewer heart-failure hospitalisations.	Mixed positive	Small trial; baseline imbalance with worse kidney function and higher NT-proBNP in the intervention group
Lim et al., 2021 [8]	Asia-Pacific region; 50 diabetes centres in 8 countries	Multinational randomised clinical trial	20,834	Adults with type 2 diabetes	JADE technology-assisted integrated diabetes care with nurse-led evaluation, personalised reports, and follow-up contacts	12 months	Diabetes-associated clinical events, HbA1c, BP, LDL-C, body weight, target attainment	Clinical events were similar between groups, but intervention participants were more likely to attain ≥2 primary diabetes targets and ≥2 key performance indices in both trial phases. Benefits were larger in low- and middle-income countries.	Positive for intermediate outcomes; null for events	Short follow-up for hard clinical events; high loss to follow-up in some phases
Provost et al., 2017 [9]	Canada; Montréal primary care cardiometabolic risk-management network	Mixed-methods implementation evaluation with pre–post quantitative analysis	1689	Adults with diabetes or hypertension	Integrated primary care cardiometabolic risk program inspired by the Chronic Care Model	12 months	Program participation, lifestyle indicators, A1c target, BP target, quality of life	Clinical target achievement improved at 12 months. Greater coordination between interdisciplinary teams and primary care physicians was associated with increased program participation and better clinical results.	Positive	Quasi-experimental implementation design; substantial program withdrawal
Aryani et al., 2016 [10]	Malaysia; primary care clinics	Cluster-controlled chronic care model study	784 enrolled; 693 analyzed	Adults with hypertension, diabetes, and/or hyperlipidemia	Chronic Care Model delivered by pharmacists, nurses, dietitians, and general practitioners	6 months	EQ-5D index, EQ-VAS, quality-of-life domains	EQ-5D index improved significantly in the intervention group from 0.92±0.10 to 0.95±0.08, while usual care showed no significant improvement. Gains were most evident in pain/discomfort and anxiety/depression domains.	Positive for QoL	Baseline group differences; quality-of-life outcome rather than primary clinical endpoint
Edelman et al., 2015 [11]	United States; community fee-for-service primary care practices	Randomised controlled trial	377	Adults with type 2 diabetes and hypertension	Nurse-led telephone behavioural case management	24 months	HbA1c, SBP, DBP, weight, physical activity, BP control	At 24 months, there were no significant differences in HbA1c, SBP, DBP, weight, physical activity, or BP-control rates between intervention and control groups.	Null	The telephone-only model may have lacked sufficient integration with routine clinical decision-making.
Santschi et al., 2021 [12]	Switzerland; ambulatory clinics and community pharmacies	Pragmatic randomised controlled trial	89	Adults with uncontrolled treated hypertension	Interprofessional team-based care involving nurses, pharmacists, and physicians	6 and 12 months	Ambulatory BP, BP control, antihypertensive medication changes	No significant BP effect at 6 months. At 12 months, daytime systolic ABPM was significantly lower in the team-based care group than usual care by −7 mmHg; diastolic BP and BP-control differences were not significant.	Mixed positive	Small sample; delayed effect; limited power for BP-control outcomes
Rosas et al., 2024 [13]	United States; federally qualified health centre	Randomised controlled trial	456	Latino adults with type 2 diabetes not using insulin	Culturally tailored integrated medical, behavioural, and diabetes education intervention	12 months	HbA1c, anxiety symptoms, depression symptoms	HbA1c and anxiety symptoms improved during the 6-month intervention and were largely maintained through 12 months. Depression symptoms showed a nonsignificant trend toward greater improvement.	Positive	Single population group; substantial missing HbA1c data
Kirsh et al., 2007 [14]	United States; Veterans Affairs primary care clinic	Quasi-experimental quality-improvement project with nonrandomized controls	44 intervention patients	Adults with diabetes at high cardiovascular risk	Shared medical appointments based on the Chronic Care Model	Approximately 10–12 months	HbA1c, LDL-C, SBP	HbA1c, LDL-C, and SBP decreased significantly after shared medical appointments. HbA1c and SBP reductions were greater than those of controls; LDL-C reduction was not significantly different between groups.	Positive for HbA1c and SBP; mixed for LDL-C	Nonrandomized design; small sample; predominantly male VA population
Mihevc et al., 2025 [15]	Slovenia; primary care centres	Multicenter randomised controlled trial	117 analyzed	Adults ≥65 years with hypertension and type 2 diabetes	mHealth home telemonitoring added to standard integrated primary care	12 months	SBP, HbA1c, DBP, fasting glucose, lipids, BMI, GFR, diabetes-related QoL	Telemonitoring significantly reduced SBP and HbA1c compared with standard care at 12 months. Secondary outcomes, including lipids, BMI, behavioural risk factors, and diabetes-related QoL, were not significantly improved.	Positive for SBP and HbA1c; limited broader effect	Small sample; older patients only; no major improvement in behavioural or quality-of-life outcomes
Kadree et al., 2025 [16]	United States; ambulatory clinic	Quality-improvement intervention with controls	134	Medicare patients with uncontrolled type 2 diabetes or hypertension	Expanded chronic care management team, including provider, nurse, pharmacist, and community health worker	4 months	HbA1c improvement, BP improvement, provider workload, and Medicare reimbursement	Diabetes control and BP improved significantly in the CCM group. Provider workload decreased, and Medicare reimbursement reached 85.5%.	Positive	Convenience sample; short follow-up; small hypertension subgroup
Gabbay et al., 2024 [17]	United States; academic cardiometabolic clinic	Retrospective cohort study	130	Adults with cardiometabolic disease	Cardiometabolic clinic integrating endocrinology and pharmacy with cardiology referral pathways	6–18 months	Medication optimization, BMI, HbA1c, LDL-C, eGFR, BP	Clinic patients had higher initiation of GLP-1 receptor agonists and SGLT2 inhibitors, greater BMI reduction, and greater HbA1c reduction than controls. LDL-C, eGFR, and BP differences were not statistically significant.	Positive for medication optimisation, BMI, and HbA1c; mixed for BP/lipids/renal markers	Retrospective design; nonrandomized referral pattern; small sample

Risk of Bias Assessment

Design-specific tools were used to evaluate risk of bias. Randomised controlled trials were evaluated using the Cochrane RoB 2 tool, and non-randomised, retrospective, implementation, and quality-improvement studies were evaluated using ROBINS-I. The included studies were heterogeneous in terms of study design, sample size, follow-up duration, intervention delivery, and outcome measurement, which led to a risk of bias. Randomised controlled trials tended to have lower selection bias, but some were open-label, had high attrition or small sample sizes and had incomplete outcome data. Nonrandomized, retrospective, implementation, and quality-improvement studies were at higher risk due to selection bias, confounding, convenience sampling, and lack of blinding (Table 3).

**Table 3:** Risk of Bias Assesment Moderate-to-high bias ratings were mainly due to real-world implementation designs, non-randomized methods, possible confounding, and lack of blinding; these factors were considered during evidence interpretation. RoB 2: Cochrane Risk of Bias 2, ROBINS-I: Risk of Bias in Non-randomised Studies of Interventions, RCT: randomized controlled trial

Study	Assessment tool	Study design	Bias due to randomisation/selection	Bias due to deviations from the intervention	Bias due to missing data	Bias in outcome measurement	Bias in reported results	Overall risk of bias
Evén et al., 2024 [7]	RoB 2	Prospective randomised open-blinded endpoint trial	Some concerns	Some concerns	Some concerns	Low	Low	Some concerns
Lim et al., 2021 [8]	RoB 2	Multinational RCT	Low	Some concerns	Moderate	Low	Low	Some concerns
Provost et al., 2017 [9]	ROBINS-I	Mixed-methods implementation evaluation with pre–post quantitative analysis.	Moderate	Moderate	Moderate	Moderate	Low	Moderate
Aryani et al., 2016 [10]	ROBINS-I	Cluster-controlled study	Moderate	Moderate	Some concerns	Moderate	Low	Moderate
Edelman et al., 2015 [11]	RoB 2	RCT	Low	Some concerns	Some concerns	Low	Low	Some concerns
Santschi et al., 2021 [12]	RoB 2	Pragmatic RCT	Low	Some concerns	Some concerns	Low	Low	Some concerns
Rosas et al., 2024 [13]	RoB 2	RCT	Low	Some concerns	Moderate	Low	Low	Some concerns
Kirsh et al., 2007 [14]	ROBINS-I	Quasi-experimental quality-improvement project	High	Moderate	Some concerns	Moderate	Moderate	High
Mihevc et al., 2025 [15]	RoB 2	Multicenter RCT	Low	Some concerns	Low	Low	Low	Some concerns
Kadree et al., 2025 [16]	ROBINS-I	Quality-improvement intervention with controls	High	Moderate	Some concerns	Moderate	Moderate	High
Gabbay et al., 2024 [17]	ROBINS-I	Retrospective cohort study	High	Moderate	Some concerns	Moderate	Moderate	High

Clinical Outcomes Across Systems-Based Interventions

**Figure 2:**
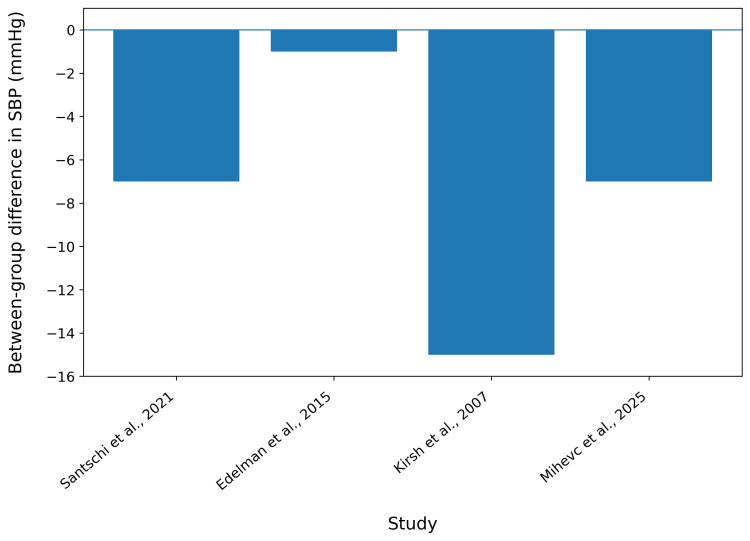
Systolic blood pressure (SBP) response to systems-based care models across included studies Sources: [11,12,14,15]

The most consistent results were seen with systems-based interventions for both SBP and glycemic control. Team-based hypertension care, shared medical appointments, expanded chronic care management, and mHealth telemonitoring were associated with blood pressure improvement. Glycemic improvement was reported for shared medical appointments, mHealth telemonitoring, culturally tailored integrated care, cardiometabolic clinic care, expanded chronic care management, and technology-assisted diabetes care. The differences in SBP between the intervention and control groups are shown in Figure 2.

**Figure 3:**
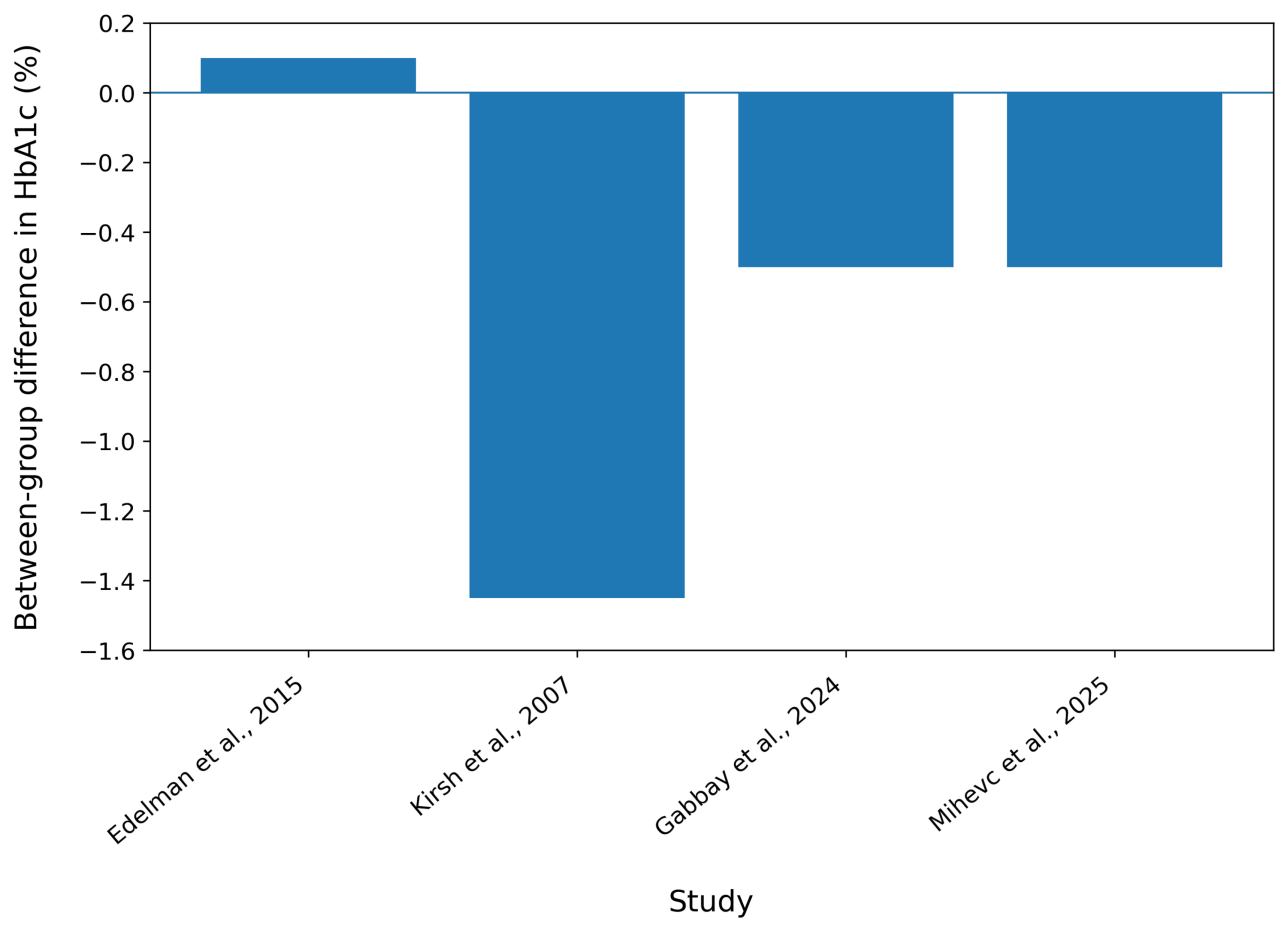
Between-group differences in HbA1c following systems-based care interventions Sources: [11,14,15,17]

Figure 3 shows the between-group difference in HbA1c (%), with most systems-based interventions associated with reductions in HbA1c and one intervention showing a slight increase.

Patient-Centred, Medication, and Health-System Outcomes

**Figure 4:**
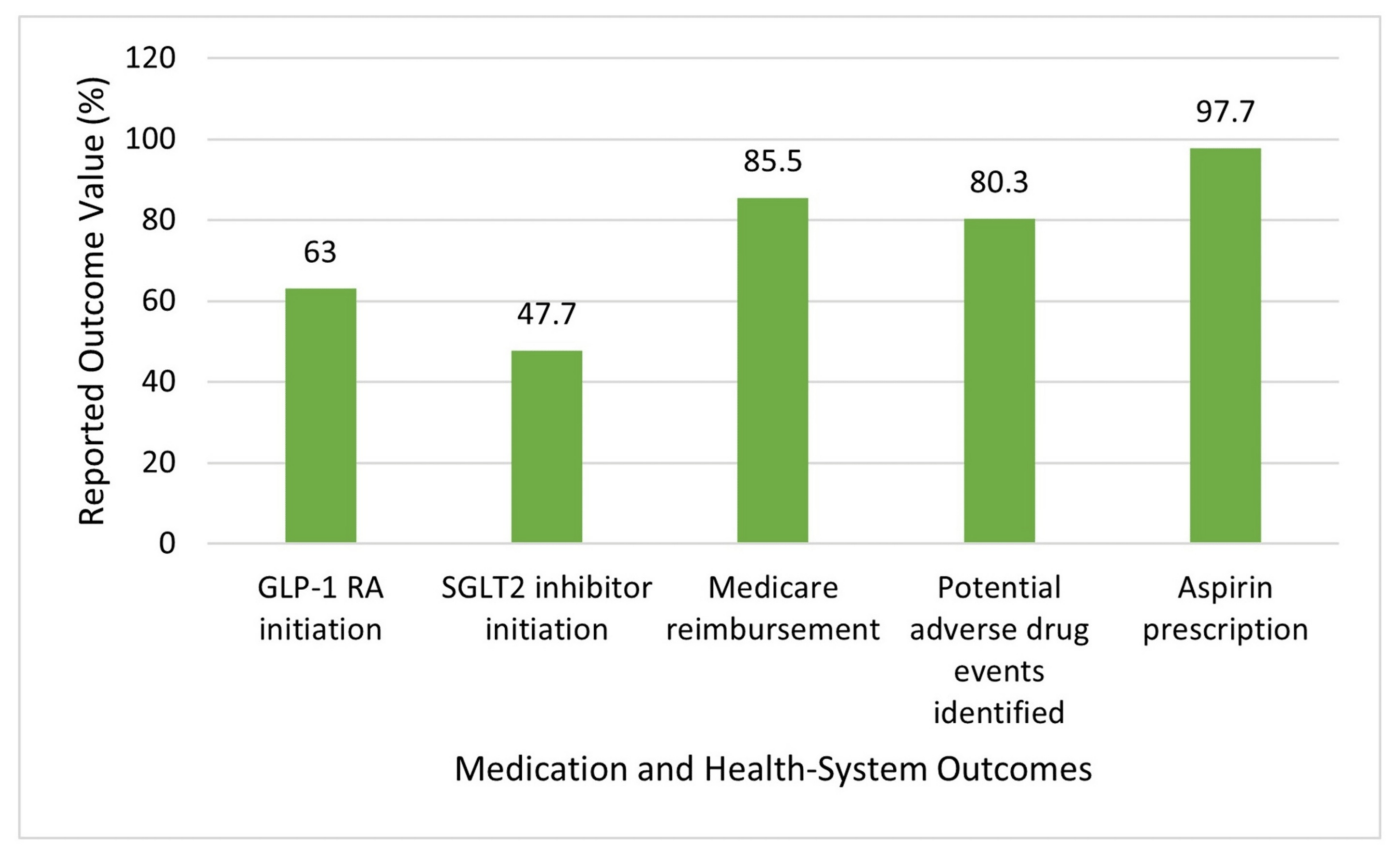
Medication and health-system outcomes reported across systems-based interventions GLP-1 RA: Glucagon-Like Peptide-1 Receptor Agonist, SGLT2 inhibitor: Sodium-Glucose Cotransporter-2 Inhibitor. Sources: [16,17]

There were several studies that demonstrated benefits other than disease-control markers. Quality of life improved in the Chronic Care Model and person-centred integrated care models. Medication optimisation was enhanced in the cardiometabolic clinic, and chronic care management models expanded. Health-system outcomes included decreased provider workload, increased reimbursement feasibility, and increased interdisciplinary team and primary care physician coordination. Figure 4 provides a summary of medication and health-system outcomes.

In general, the greatest advantages were seen with systems-based approaches, especially for intermediate cardiometabolic outcomes, such as SBP and HbA1c. Other benefits of quality of life, medication optimisation, and health-system performance were also reported, but evidence for the reduction of major cardiovascular, renal, or mortality outcomes was limited.

Discussion

The results of this systematic review identified that systems-based models of adult cardiometabolic and chronic disease management were most consistently linked to improvements in intermediate clinical outcomes (SBP and HbA1c). Overall, interventions that went beyond one-on-one clinician-patient interactions and included structured monitoring, multidisciplinary input, medication review, behavioural support, patient education, and coordinated follow-up seemed more likely to be effective. This interpretation is supported by the current standards of diabetes care, which include population health, team-based care, patient-centred decision-making and timely therapeutic adjustment as important elements of chronic disease management [21].

Blood pressure outcomes indicated that systems-based interventions may be particularly effective if they provide multiple opportunities for blood pressure to be measured, feedback provided, and therapeutic adjustments made. Evidence for improvements was greater in models with team-based hypertension care, shared medical appointments, mHealth telemonitoring and extended chronic care management. These findings suggest that hypertension control is not only a function of drug availability, but also depends on follow-up systems that can detect uncontrolled blood pressure, ensure adherence and enable the clinician to step up treatment, if necessary. This is clinically significant as cardiometabolic patients frequently have multiple co-existing risk factors; blood pressure management needs to be a part of diabetes, kidney disease, and cardiovascular risk management, and not stand alone.

Systems-based approaches also showed an advantage for glycemic outcomes, especially when interventions involved structured assessment, active patient involvement, and medication optimisation. Models that included telemonitoring, integrated diabetes care, shared medical appointments, culturally tailored education, cardiometabolic clinics, or expanded chronic care management showed more improvements in HbA1c. The findings contribute to larger initiatives to redesign care for C-R-M conditions at the system level for the purposes of improving guideline-directed therapy. The “Beyond Diabetes” program was a programmatic strategy to enhance guideline-directed therapy for C-R-M conditions that focused on clinical pathways, provider education, and systematic identification of gaps in therapy [22,23]. The current review also indicates that the more care systems are involved in organising monitoring, education, prescribing and follow-up, the more likely glycemic improvement is.

One mechanism that was identified as being important with respect to systems-based care and cardiometabolic outcomes is medication optimisation. Several studies included found that pharmacists, nurses, community health workers or speciality teams added to the care pathway resulted in better medication initiation, medication review, or adverse drug event identification. A broadened chronic care management model involving a provider, nurse, pharmacist, and community health worker was associated with improved diabetes and blood pressure outcomes, reduced provider workload, and fiscal sustainability through Medicare reimbursement [16]. It is applicable for routine practice because chronic disease management increasingly requires medication reconciliation, adherence support, safety monitoring and patient education, which may be more than physicians or nurses can do in a physician-nurse model.

The results were also a testament to the clinical benefits of cardiometabolic collaboration. Chang et al. proposed that there are care gaps in diabetes and cardiovascular disease that could be resolved by bringing the specialities closer together and by implementing more integrated approaches to cardiometabolic care [24,25]. This review reinforces that view by demonstrating that clinic-based and team-based formats can help increase the use of evidence-based cardiometabolic interventions and improve care coordination across complex profiles of risk. This collaboration is especially significant in the context of newer medications such as glucagon-like peptide-1 (GLP-1) receptor agonists and sodium-glucose cotransporter 2 (SGLT2) inhibitors, which must be carefully selected, counselled, monitored and followed up in all areas of diabetes, cardiovascular, renal and metabolic.

Patient-centred and health-system outcomes were also important. Across the studies included, quality of life, anxiety symptoms, self-management, provider workload, reimbursement feasibility, and medication safety were reported. The outcomes are relevant due to the fact that management of cardiometabolic disease does not only involve biochemical control. Multimorbidity is associated with treatment burden, fragmented appointments, psychological stress and poor maintenance of lifestyle change. Systems-based care can alleviate some of this burden by streamlining services, enhancing communication, and providing more predictable follow-up. This is reinforced by the new cardiovascular-kidney-metabolic paradigm, which considers metabolic dysfunction as a chronic multisystemic disease process and not a collection of individual diagnoses [26].

Results were less consistent with regard to lipid outcomes, body mass index, behavioural risk factors and major cardiovascular or renal events. This can be due to varying levels of intervention intensity across studies, differing sample sizes, follow-up durations, baseline risk, and outcome measures. Intermediate measures (SBP, HbA1c) may demonstrate improvement earlier than cardiovascular, renal or mortality measures, which may take longer follow-up and larger populations to demonstrate. The lack of consistent hard-outcome benefits does not mean that systems-based care is ineffective, but rather that longer pragmatic studies with common clinical endpoints are needed.

Findings have implications for primary care and speciality care organisations. Säemann et al. advocated for more robust C-R-M guidance for primary care physicians, highlighting the need for practical guidance for implementation that includes risk recognition, initiation of treatment, and well-coordinated follow-up [24]. The evidence brought together in this review backs that direction. Systems-based care seems most effective when it links primary care, the pharmacy, nursing, behavioural support services, community health workers, and speciality services into a collaborative process of care.

In general, systems-based strategies seem to be most beneficial when they incorporate multidisciplinary skills, structured monitoring, medication optimisation, behavioural support, patient education, and coordinated follow-up. These models are designed to help move away from visit-based, reactive, disease-specific care to proactive, cardiometabolic risk care as a unified clinical issue. Future research is needed with larger pragmatic designs, longer follow-up, standardised outcome definitions, and cost-effectiveness analyses to evaluate if better intermediate outcomes lead to sustained improvements in cardiovascular outcomes, renal progression, hospitalisation, and mortality.

Limitations and Future Directions

There are some limitations to this review. The studies included varied widely in study design, population, type of intervention, clinical setting, length of follow-up and definition of outcomes, making direct comparisons difficult and precluding meta-analysis. Several of the studies were nonrandomized, retrospective, or quality-improvement studies, which led to the potential for selection bias, confounding, and failure to adequately control for baseline differences. Some of these randomised trials were open-label, lacked outcome data or were small. Only English-language full-text studies were included, which may have resulted in the exclusion of relevant evidence.

Future studies should be conducted with large, multicenter pragmatic trials with longer follow-up to assess whether improved HbA1c, SBP and medication optimisation, and improved quality of life, are associated with fewer cardiovascular events, renal progression, hospitalisation, or death. Standardised outcome definitions, consistent reporting of intervention components, and formal cost-effectiveness analyses should be included in studies. Additional evidence is also needed to determine which system-based components are most effective, sustainable, and can be scaled to primary care, speciality care, and underserved populations.

## Conclusions

The results of this systematic review suggest that systems-based strategies may improve adult chronic and cardiometabolic disease management, particularly intermediate outcomes such as HbA1c and systolic blood pressure. Multidisciplinary care, structured monitoring, medication optimisation, patient education, telemonitoring, shared medical appointments, and coordinated follow-up appear most useful for strengthening care processes and risk-factor control. This review contributes to the existing literature by synthesising evidence across clinical, patient-centred, medication-related, and health-system outcomes rather than focusing only on single-disease clinical endpoints. Its findings support the value of integrated care models for adults with cardiometabolic multimorbidity, while also showing that evidence remains less consistent for lipid control, body mass index, behavioural risk factors, cardiovascular events, renal events, and mortality. Future studies should use larger pragmatic designs, longer follow-up, standardised outcome measures, and economic evaluations to determine whether systems-based care produces sustained clinical benefit and improved health-system efficiency.
